# Liver-Targeting of Interferon-Alpha with Tissue-Specific Domain Antibodies

**DOI:** 10.1371/journal.pone.0057263

**Published:** 2013-02-25

**Authors:** Edward Coulstock, Jane Sosabowski, Milan Ovečka, Rob Prince, Laura Goodall, Clare Mudd, Armin Sepp, Marie Davies, Julie Foster, Jerome Burnet, Gráinne Dunlevy, Adam Walker

**Affiliations:** 1 Innovation Biopharm Discovery Unit, Biopharm R&D, GlaxoSmithKline, Cambridge, United Kingdom; 2 Centre for Molecular Oncology, Barts Cancer Institute, Queen Mary University of London, London, United Kingdom; Genentech, United States of America

## Abstract

Interferon alpha (IFNα) is used for the treatment of hepatitis C infection and whilst efficacious it is associated with multiple adverse events including reduced leukocyte, erythrocyte, and platelet counts, fatigue, and depression. These events are most likely caused by systemic exposure to interferon. We therefore hypothesise that targeting the therapeutic directly to the intended site of action in the liver would reduce exposure in blood and peripheral tissue and hence improve the safety and tolerability of IFNα therapy. We genetically fused IFN to a domain antibody (dAb) specific to a hepatocyte restricted antigen, asialoglycoprotein receptor (ASGPR). Our results show that the murine IFNα2 homolog (mIFNα2) fused to an ASGPR specific dAb, termed DOM26h-196-61, could be expressed in mammalian tissue culture systems and retains the desirable biophysical properties and activity of both fusion partners when measured *in vitro*. Furthermore a clear increase in *in vivo* targeting of the liver by mIFNα2-ASGPR dAb fusion protein, compared to that observed with either unfused mIFNα2 or mIFNα2 fused to an isotype control dAb V_H_D2 (which does not bind ASGPR) was demonstrated using microSPECT imaging. We suggest that these findings may be applicable in the development of a liver-targeted human IFN molecule with improved safety and patient compliance in comparison to the current standard of care, which could ultimately be used as a treatment for human hepatitis virus infections.

## Introduction

The current standard of care for hepatitis C virus (HCV) infection is treatment with pegylated IFN alpha, (Pegasys® and Pegintron®) in combination with the nucleoside analogue Ribavirin [1], [2]. The potent anti-viral, anti-proliferative and immunomodulatory mechanisms of the type I interferons, a class of cytokines to which IFNα belongs, are well documented [3]. Whilst clearly efficacious, the systemic delivery of IFNα not only generates an anti-viral response in the liver, but also results in leukocyte activation in the blood leading to adverse responses to the therapy including cytokine release, flu-like symptoms and depression. These side-effects can be severe which leads to a significant proportion of patients discontinuing treatment [4], [5], [6].

The targeting of bioactive molecules to tissues is an attractive concept and in particular may offer multiple benefits in the treatment of HCV with IFNα. The perceived benefits are two-fold, namely increasing the local concentration of a therapeutic compound at the required site of action, potentially retaining efficacy with a reduced dose, and reducing undesired activity of a therapeutic in non-target tissues, potentially improving safety and tolerability. The application of this concept in multiple disease indications has been investigated using a wide range of methodologies, for example site-specific delivery of cytotoxic drugs for cancer therapy [7], [8], liposomal delivery of antigens in vaccine development [9] and the targeting of blood-brain barrier (BBB) receptors to facilitate transfer of biopharmaceuticals from the blood into the brain parenchyma [10].

Viral replication in HCV infection occurs predominantly in the liver. Asialoglycoprotein receptor (ASGPR) is a cell surface receptor expressed exclusively in hepatic parenchymal cells [11]. ASGPR is a C-type (calcium dependent) lectin composed of two transmembrane glycoprotein subunits, termed H1 and H2. The aglycosyl H1 and H2 subunits are approximately 35 and 33 kDa in size respectively, though purified ASGPR protein subunits are significantly larger due to post-translational modification. ASGPR mediates endocytosis of plasma glycoproteins that have exposed terminal galactose residues from which terminal sialic residues have been removed [12]. In addition, ASGPR has also been linked to the entry of HCV into hepatocytes [13]. Despite reports of potential extra hepatic expression in human kidney [14], thyroid [15] and activated T cells [16], ASGPR has been exploited in the targeting of therapeutic molecules to the liver. For example, ASGPR-targeted nanoparticles loaded with cytotoxic agents such as paclitaxel result in enhanced cell killing activity against ASGPR-positive cell lines when compared with free paclitaxel [17]. ASGPR-directed nanoparticles have also been used to deliver transgenes and antisense oligonucleotides to ASGPR-expressing primary hepatocytes and cell lines [18], [19]. *In vivo* radioiodinated copolymers with ASGPR binding activity accumulate in the liver following intravenous administration in rats [20]. In a study conducted by Peng *et al.*, systemic delivery of the apoptin gene, which selectively induces apoptosis in malignant cells, linked to asialoglycoprotein resulted in specific delivery to ASGPR-positive HepG2 derived tumors xenografted in SCID mice and significant tumour regression. By contrast ASGPR-apoptin transgene conjugates were not able to induce tumour regression in non-hepatocyte derived A549 xenografted animals [21].

Compelling evidence for the potential application of ASGPR-mediated hepatic delivery in improving antiviral efficacy of type I interferons is provided in a study by Eto and Takahashi. Following enzymatic removal of terminal sialic acid residues from the N-linked oligosaccharide chain of human interferon beta (IFNβ), the investigators were able to demonstrate enhanced interferon signaling activity and inhibition of viral replication in HBV transfected HepG2 cells compared to the unmodified form of the protein [22]. This enhanced antiviral activity was presumably due to ASGPR binding, as it could be partially inhibited by natural ASGPR ligands such as asialofetuin. Significantly enhanced *in vivo* antiviral efficacy of murine asialo-IFNβ, compared with that of the unmodified protein, was also shown in HBV transfected BALB/c athymic nude mice.

In this study, using phage display technology we generated a dAb specific for ASGPR and genetically fused it to IFNα. The small size of dAbs (11–15 kDa) coupled with their high affinity for their respective antigen can help preserve the activity of fusion partners so makes their use attractive [23], [24], [25]. We show that the IFNα-ASGPR dAb fusion protein can be expressed in mammalian cells, that it binds to ASGPR expressed on liver cell lines and retains cytokine activity. Furthermore, using SPECT imaging we show that the fusion specifically targets the liver suggesting that this approach may have therapeutic application and ultimately lead to a reduction in adverse events associated with systemic delivery of IFNα.

## Materials and Methods

### Antigen and mIFNα2 protein generation

Human and murine ASGPR-H1 ectodomains and mIFNα2 were generated as His_(6)_-tagged inserts via PCR and cloned into pDOM50, a derivative of the pTT5 HEK293E expression vector (National Research Council, Canada) using BamHI/HindIII restriction sites. Protein was expressed in HEK293 cells and secreted into the culture supernatant [26]. Expressed protein was then purified on Ni-NTA resin (Qiagen) according to manufacturer's instructions. Purified proteins were dialysed into Dulbecco's PBS.

### Selection and Isolation of ASGPR specific dAbs by phage display

Human ASGPR antigen was passively coated on immunotubes (Nunc) overnight at 1 mg/ml in Tris-HCl buffered saline (TBS; 50 mM Tris-HCl, 150 mM NaCl, pH 8) supplemented with 5 mM CaCl_2_ (TBS/Ca^2+^). After coating with antigen, tubes were blocked by addition of 2% (w/v) Marvel non-fat milk in TBS supplemented with 5 mM CaCl_2_ (MTBS/Ca^2+^). Selections were carried out using phage libraries displaying antibody single variable domains. Library aliquots were incubated with antigen-coated immunotubes in MTBS/Ca^2+^ before washing tubes with TBS/Ca^2+^. Bound phage was then eluted with 1 mg/ml trypsin. Following selection, eluted phage was used to infect log phase TG1 *E.coli* and then infected cells were plated onto LB agar supplemented with 15 µg/ml tetracycline. Cells infected with the phage were then grown up in 2× yeast tryptone (2× YT) medium (supplemented with tetracycline) overnight at 37°C before precipitation of phage from the culture supernatant using chilled PEG-NaCl (20% (v/v) polyethylene glycol 8000, 2.5 M NaCl) and used in a second round of selection as above, using antigen coated at 0.2 mg/ml. Precipitated phage from this second round of selection was subsequently used in a third round of selection using antigen coated at 0.04 mg/ml.

### Screening selection outputs for ASGPR specific dAbs

After three rounds of selection, dAb genes from each library pool were subcloned from the phage vector using the restriction enzymes SalI and NotI. The enriched dAb genes were ligated into the corresponding sites in pDOM10, a pUC119-based vector, in order to facilitate soluble expression of dAb proteins with a C-terminal FLAG epitope tag into the periplasm and culture supernatant of *E. coli* TOP10 cells (Invitrogen) transformed with the dAb expression construct. Transformants were grown overnight on LB agar plates supplemented with 100 µg/ml carbenicillin and 5% (w/v) glucose.

Individual colonies were then picked into 96-well plates containing 100 µl/well 2× YT medium supplemented with 100 µg/ml carbenicillin and grown at 37°C for 4 hours with shaking at 250 rpm. Protein expression was induced by addition of 100 µl per well 2× YT medium supplemented with 100 µg/ml carbenicillin and 0.1 mM IPTG, with overnight incubation using the same conditions.

The antigen binding of individual dAb clones was assessed by ELISA. Human ASGPR antigen was coated at 1 µg/ml onto a Maxisorp (NUNC) plate overnight at 4°C. The plate was then blocked with 2% (v/v) Tween 20 in TBS/Ca^2+^, followed by incubation with dAb supernatant diluted 1∶1 with 0.1% (v/v) Tween 20-TBS/Ca^2+^, followed by detection with 1∶5000 anti-FLAG (M2)-HRP (Sigma-Aldrich). All steps after blocking were carried out at room temperature. The binding of the dAb supernatant to a His_(6)_-tagged glycosylated irrelevant control antigen was also analysed.

### Expression and purification of dAb proteins

FLAG-tagged dAbs were expressed in TOP10 *E. coli* following growth in TB OnEx auto induction medium (Novagen) supplemented with 100 µg/ml carbenicillin at 30°C for 72 hrs. Expressed protein was purified from clarified culture supernatant using protein A coupled to NHS streamline resin (GE Healthcare). Briefly, proteins were batch-bound to resin for 4 hrs at room temperature before washing with 10 column volumes of 25 mM Na Acetate, pH 6. Bound protein was eluted with 4 CV 25 mM Na Acetate, pH 3, and then subsequently neutralised with 1/10^th^ vol. 1 M Na Acetate, pH 6.

### Emulsion Based Affinity maturation of ASGPR specific dAb DOM26h-196

Affinity maturation of dAb clone DOM26h-196 was carried out by diversification of CDR regions using doped oligos where the diversified positions contained 85% parent base and an equimolar mix of the remaining three bases. Briefly the amplification products for individual CDR libraries were purified by gel electrophoresis, mixed in equal ratios, spliced by ‘splicing of overlapping extension’ (SOE) PCR and re-amplified by PCR. Spliced and amplified libraries were thereafter cut with SalI and NotI enzymes and ligated into pIE2a2A vector. The *in vitro* expression construct was PCR amplified from the ligation and the libraries for CDR1, CDR2 and CDR3 were thereafter pooled for selection. Nine rounds of selection were carried out in total. In the first round of selection 2.5×10^9^ molecules of library were compartmentalised in 1 ml of emulsion, in the subsequent eight rounds 5×10^8^ DNA molecules per reaction were used. Affinity capture of protein-DNA complexes was carried out using human ASGPR antigen biotinylated with NHS-LC-biotin (Pierce, according to manufacturer's protocol). M280 Streptavidin Dynabeads at 3×10^7^ beads per reaction (Invitrogen) were used throughout to capture ligand-dAb-DNA complexes. Following the final round of selection, the amplified DNA was cut with SalI/NotI enzymes, cloned into pDOM10 vector and transformed into Mach1 Chemically competent cells (Invitrogen). Selection outputs were expressed as described above and assayed for antigen-binding activity by BIAcore using both human and mouse ASGPR. Affinity maturation screening identified beneficial mutations in all three CDRs of clone DOM26h-196, suggesting the possibility of further improvement by CDR shuffling and screening. CDR1, 2 and 3 sequences from several improved clones were amplified by PCR before purifying by gel electrophoresis, mixing in equal ratios and splicing by SOE PCR. Spliced PCR fragments were cloned into pDOM10 expression vector and transformed into Mach1 Chemically competent cells (Invitrogen). 96 transformants were picked and used to express/purify dAb protein as described above. All 96 clones were assayed for antigen-binding activity by BIAcore, and from this screen clone DOM26h-196-61 was identified.

### Flow cytometry

Flow cytometry data was generated using the ASGPR positive human hepatocarcinoma derived cell line Huh7, primary human hepatocytes (Celsis IVT) and the ASGPR negative monocyte derived cell line U937 (American Type Culture Collection, ATCC number CRL-1593.2). Adherent Huh7 cells were harvested using an enzyme-free cell dissociation buffer (Gibco) and washed in PBS containing Ca2+ and Mg2+ supplemented with 10% (v/v) pooled human serum (Sigma) (FACS buffer). U937 cells were harvested from culture & washed in FACS buffer & frozen primary hepatocytes were revived from storage immediately prior to the assay. All cells were seeded into 96 well v- bottomed microtitre plates (Nunc) at a concentration of 1×10^5^ cells per well and incubated for 45 minutes at 4°C with the appropriate concentration of dAb or V_H_D2 isotype control molecule. The cells were then washed with FACS buffer and incubated for one hour at 4°C with a mouse monoclonal antibody specific to human V_H_ domains (anti-V_H_) for 45 minutes at 4°C. Following the incubation, cells were washed as before and were then incubated with an Alexa-647 conjugated goat anti-mouse pAb (Molecular Probes). Cells were then washed as before and resuspended in 100 µl PBS containing 1 µM Sytox®Blue dead cell exclusion dye (Molecular Probes). Fluorescence intensity in the APC channel was determined using a BD FACS Canto II flow cytometer.

### Cloning and expression of mIFNα2-dAb fusion proteins

mIFNα2-dAb fusions were generated by SOE PCR and the inserts cloned into pDOM50, again using BamHI/HindIII. The mIFNα2-dAb fusions were expressed in HEK293 cells as previously described and then purified on protein A streamline or NiNTA resins as described above.

### Surface plasmon resonance assay

BIAcore™ SA chip (Series S Sensor Chip SA Certified, GE Healthcare Bio-Sciences AB) was coated with biotinylated recombinant mouse ASGPR1. One flow cell (Fc1) was used as a reference flow cell. HBS-P+ buffer (10 mM HEPES, 150 mM NaCl, 0.05% Surfactant P20, pH 7.4) was used as running buffer. The sample compartment of the instrument was kept at 4°C throughout the experiment. The data collection rate was 10 Hz. A concentration series of molecules under investigation, prepared in running buffer, were injected over the surface of the chip at 50 µl/min flow rate at 25°C. The injection time was 90 s and the dissociation time was 120 s. Regeneration of the chip surface between injections (back to baseline) was carried out using 10 mM glycine pH 2.0 solution.

### B16-Blue™ interferon activity assay

To confirm activity of the mIFNα2 arm of the fusion protein, B16-Blue™ IFNα/β cells (InvivoGen), stably transfected with a SEAP reporter gene under the control of the ISG54 promoter enhanced by a multimeric ISRE, were seeded at 5×10^4^ cells/well in 50 µl of assay media (RPMI1640 supplemented with 10% FBS (v/v)) in 96-well microtitre plates. Test molecules, over a dose range up to 300 nM, were added in a 50 µl volume of assay media to the plates in quadruplicate and the plates incubated in a 5% CO_2_ humidified atmosphere at 37°C. After 20 hours 40 µl of the culture supernatant was then transferred to fresh 96-well microtitre plate and 160 µl of QUANTI-Blue™ SEAP visualization reagent (made according to manufacturer's instructions) was added to each well. The plates were left at room temperature until an absorbance at 640 nm of 1.0–1.5 for the mIFNα2 control had been reached. The absorbance was read using an M5e plate reader (Spectramax). Data was analysed using GraphPad Prism software (Graphpad Software Inc.).

### Conjugation of V_H_D2 isotype control dAb with maleimide-DOTA

All buffers used for preparation, purification and radiolabelling of dAb/mIFNα2 conjugates were pre-treated with Chelex-100 resin (Bio-Rad). V_H_D2 isotype control dAb was dialysed into 0.25 M HEPES, pH 7. The dialysed solution was treated with TCEP at a final concentration of 5 mM for 30 min followed by the addition of a 30-fold excess of maleimide-DOTA (Macrocyclics, Inc.). The reaction mixture was then left at room temperature overnight in the dark and then applied to protein A streamline resin equilibrated in HEPES, pH 7 before washing with 30 ml HEPES, pH 7 and elution in 0.25 ml fractions of 0.1 M glycine/HCl pH 2) into tubes containing ammonium acetate (final concentration and pH of fractions was 0.46 M ammonium acetate, pH 5). The conjugated protein was analysed by ESI-MS to confirm conjugation.

### Conjugation of DOM26h-196-61, mIFNα2 and mIFNα2-dAbs with NHS-DOTA

DOM26h-196-61 and mIFNα2 were dialysed into PBS (PAA Laboratories GmbH, Pasching, Austria) while the mIFNα2-dAb fusions were dialysed into 25 mM Na Acetate solution, pH 8. NHS-DOTA (Macrocyclics, Inc.) was then added in a 4-fold molar excess and reacted overnight at room temperature. Conjugation solutions (except for mIFNα2) were then applied to protein A columns equilibrated in, pH 7.4, before washing with PBS, pH 7.4 and elution in 0.5 ml fractions of 0.1 M glycine/HCl, pH 2, into tubes containing ammonium acetate (final formulation of fractions was 0.46 M ammonium acetate, pH 5). In the case of mIFNα2, the conjugation reaction was purified by dialysis against 0.2 M ammonium acetate, pH 5.5. Conjugated proteins were analysed by ESI-MS to confirm conjugation. Surface plasmon resonance and B16-Blue™ assays were performed to assess ASGPR binding and mIFNα2 activity, respectively.

### Radiolabelling of DOTA-protein conjugates and radiochemical analysis

The general radiolabelling protocol was as follows; 40–60 µl (26–50 MBq) of ^111^InCl_3_ in 0.05 M HCl, 8–12 µl (1/5th the volume) of 1 M ammonium acetate, pH 4.5–5.5 (or in the case of IFN-DOM26h-196-61, 120 µl 0.1 M MES, pH 5.1) and 12.5–92.5 µg of protein was added to a low protein binding 1.5 ml polypropylene tube. The solution was heated to 40°C for 1.5–2.5 h and quenched with 0.1 M EDTA solution (1/20^th^ reaction volume). Radiochemical purity was determined using size exclusion HPLC (SEC) using a YMC-Pack Diol-60 column (YMC Europe GmbH, Germany), 300×8.0 mm i.d., S-5 µm with a mobile phase of 200 mM phosphate buffer, pH 6.8 and thin layer chromatography (TLC), iTLC-SG (Varian Inc., CA) eluted with 0.1 M ammonium acetate, 50 mM EDTA, pH 5.5 or iTLC-SG (pre-soaked in 2% (w/v) BSA in water and dried at 37°C) eluted with 1∶2∶5 35% ammonia∶ethanol∶water. The reaction mixture was then diluted with 0.1% (w/v) bovine serum albumin in PBS (BSA/PBS) followed by filtration through a 0.22 µm filter.

### MicroSPECT/CT imaging

Radiolabeled dAb or mIFNα2-dAb fusion proteins diluted with BSA/PBS (10–17 MBq) were injected intravenously via the tail vein into a single female BALB/c mouse (except in the case of ^111^In-DOTA-mIFNα2 where a female beige SCID was used). Whole body SPECT images were obtained under isoflurane anaesthesia at 3, 24 and 72-hrs (45-min each) using a Bioscan NanoSPECT/CT four-head camera (Bioscan Inc. Washington) fitted with 2 mm pinhole collimators in helical scanning mode (20 projections, 45-min scan) and CT images with a 45 kVP X-ray source. Images were reconstructed using InVivoScope software (Bioscan Inc).

### Biodistribution studies in BALB/c mice

Radiolabel dAbs or mIFNα2-dAb fusion proteins diluted with BSA/PBS (0.15–0.5 MBq, radiolabelling efficiency corrected dose) were injected into female BALB/c mice (*n* = 4) via the tail vein. After 3 hours, the animals were terminated and tissues and organs of interest were collected, weighed and counted along with appropriate standards in a gamma counter (1282 Compugamma CS, LKB Wallac).

## Results

### Selections and affinity maturation

**Figure 1:**
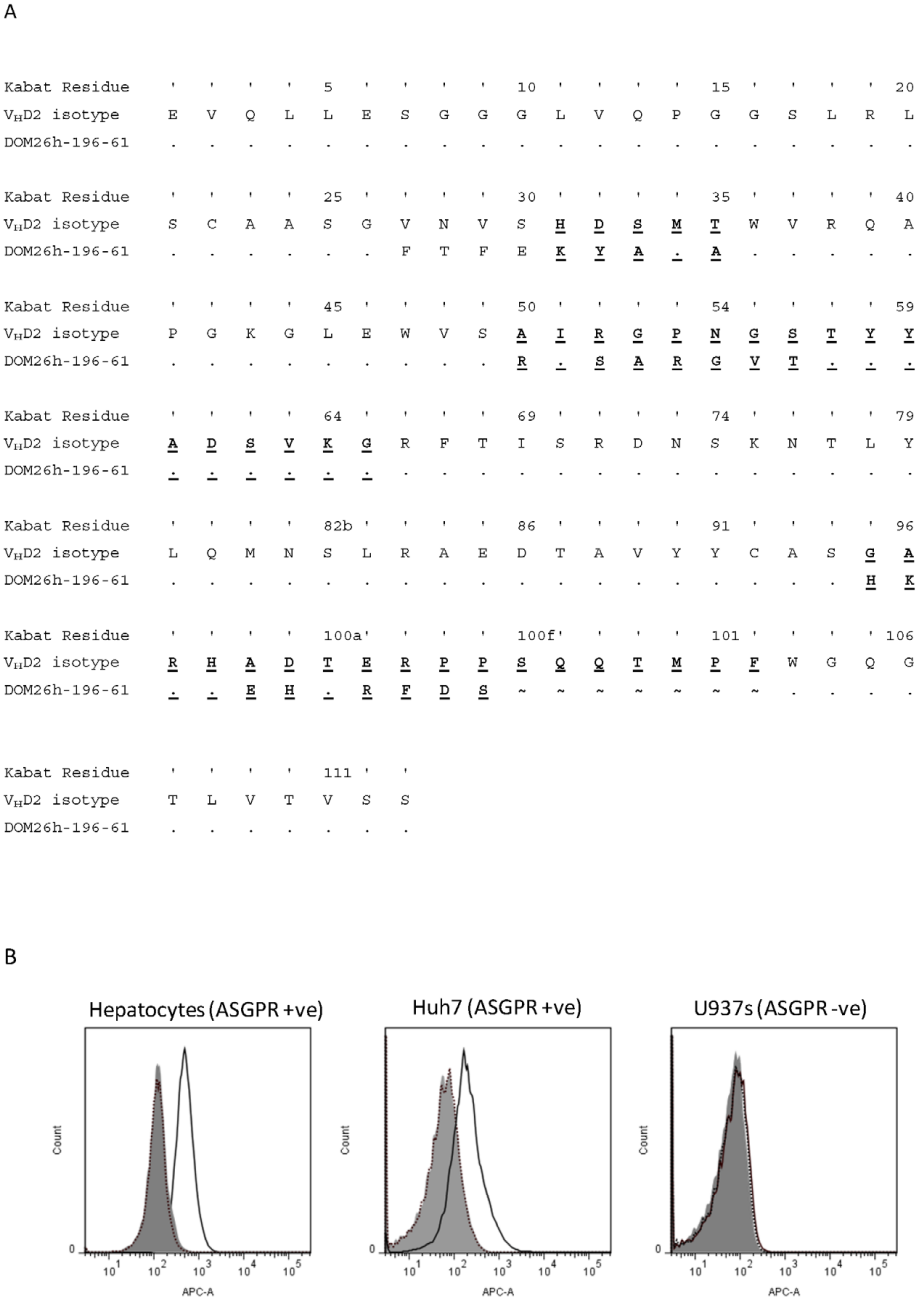
A – Sequence of DOM26h-196-61. Alignment of the primary sequence of the ASGPR specific dAb DOM26h-196-61 and the V_H_D2 isotype control sequence. DOM26h-196-61 residues identical to the V_H_D2 isotype control sequence are represented by ‘.’ Residue numbering was determined by the method of Kabat [34], with the residues contained within the three complementary determining regions (CDRs) underlined and in bold. In CDR3 of DOM26h-196-61, the symbol ‘∼’ has been used to facilitate alignment but does not represent a residue. **B** – Flow cytometry of liver targeting dAbs. ASGPR specific DOM26h-196-61 dAb (solid line) and V_H_D2 isotype control dAb (dotted line) were tested for binding to ASGPR positive primary human hepatocytes and the human hepatocarcinoma-derived cell line Huh7 by flow cytometry. Binding of DOM26h-196-61 to the ASGPR negative human cell line U937 is also shown for comparison. Detection of bound dAbs was demonstrated using a mouse monoclonal antibody specific for human V_H_ domains and an Alexa 647 conjugated goat anti-mouse pAb. Staining of cells in the absence of dAb is also shown for comparison (shaded histograms).

A series of ASGPR specific dAbs were selected from phage libraries using recombinant ASGPR H1 subunit purified from HEK293 culture supernatant. One clone, DOM26h-196, was chosen for further affinity improvements based on expression, selectivity/affinity for antigen and solution state properties (namely monomeric in solution as determined by size exclusion chromatography and melting temperature >55°C as determined by differential scanning calorimetry). Following affinity maturation, several DOM26h-196 derived dAbs were obtained that showed specific binding to ASGPR H1 subunit by surface plasmon resonance and ELISA (data not shown). DOM26h-196-61 dAb (amino acid sequence shown in Figure 1A) showed high affinity binding to the human ASGPR-H1 subunit (K*_D_* = 1 nM), as well as high affinity binding to the murine antigen (K*_D_* = 4 nM). Due to its human/mouse cross-reactivity this dAb was chosen as the targeting fusion partner for the IFN payload. The isotype control dAb, V_H_D2, in contrast demonstrated no binding activity to human or murine ASGPR-H1.

When tested by flow cytometry for binding to ASGPR positive and negative cell lines DOM26h-196-61 bound to the ASGPR positive cell line Huh7, with negligible binding to ASGPR negative U937 cell line observed. In addition to binding ASGPR positive cell lines DOM26h-196-61 also bound to primary human hepatocytes (Figure 1B). This strongly suggests that the ASGPR specific dAb DOM26h-196-61 specifically binds to native ASGPR expressed on the surface of hepatocytes and hepatocyte derived cell lines. As expected the V_H_D2 isotype control dAb did not bind to any of the cells tested.

### Expression and purification of mIFNα2-dAb fusion proteins

**Figure 2:**
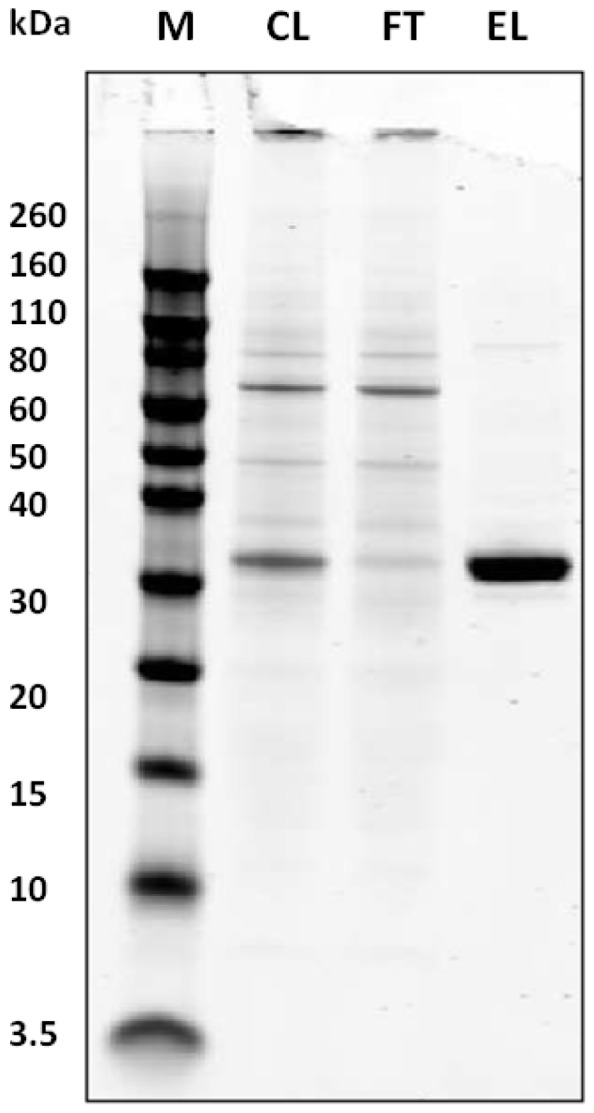
SDS-PAGE analysis of mIFNα2-dAb fusion protein purification. Purification on protein A Streamline resin from clarified cell culture supernatant results in a single band of the expected molecular mass of approximately 33 KDa. CL = clarified supernatant, FT = flowthrough fraction, EL = eluted fraction.

The mIFNα2-dAb fusion proteins were expressed in the pTT5/HEK293 mammalian expression system with expression levels comparable to those of hIFNα2b- AlbudAb™ fusions described previously [28]. A simple, one step purification procedure using protein A streamline resin provided pure material, as determined by SDS-PAGE (Figure 2), with typical yields of 20 mg protein obtained per litre of culture supernatant.

**Figure 3:**
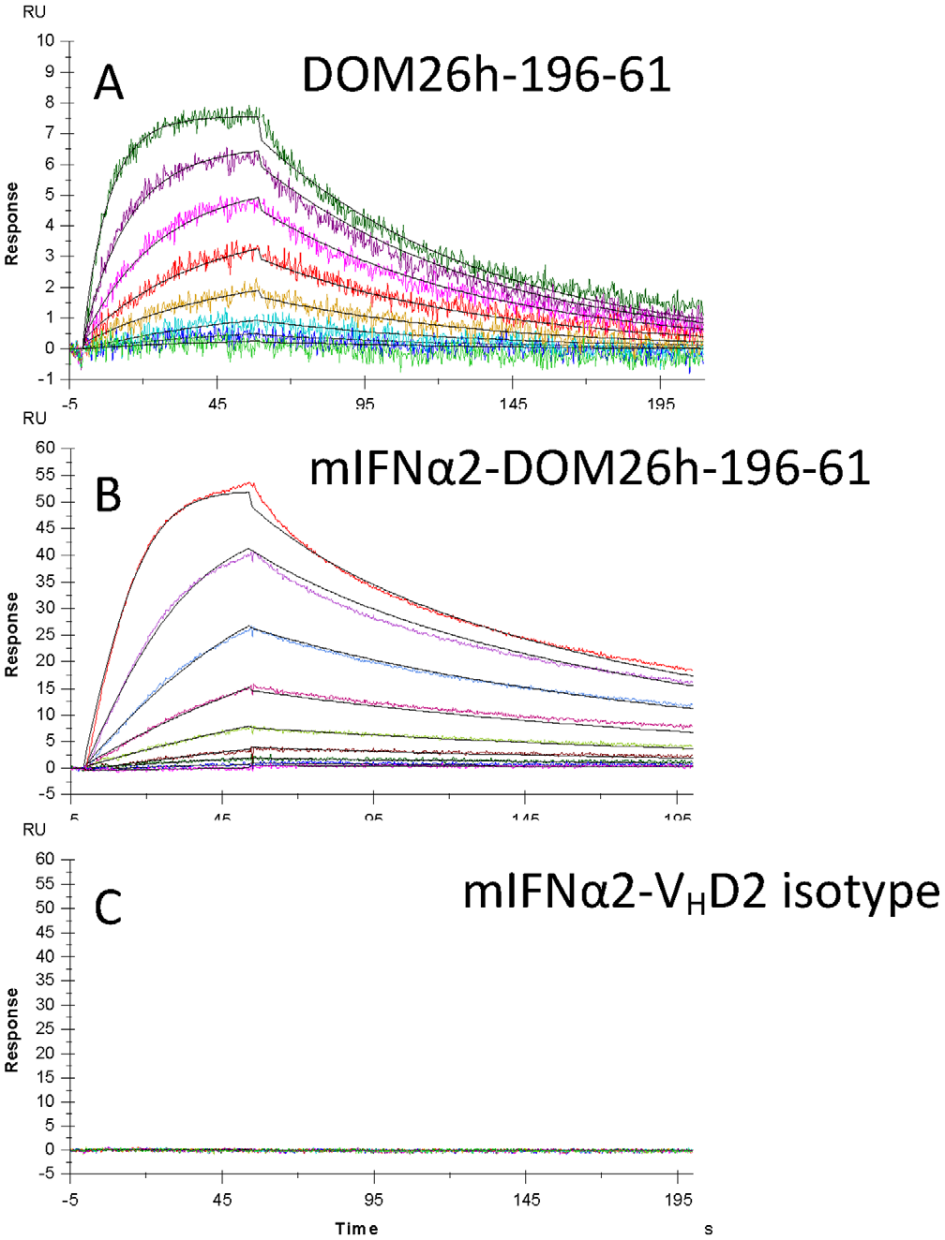
Surface plasmon resonance analysis of ASGPR specific dAbs and mIFNα2-dAb fusion proteins. Murine ASGPR H1 antigen immobilised on CM5 chip surface was used to analyse binding kinetics of DOM26h-196-61 and mIFNα2-DOM26h-196-61 injected over the chip surface at a constant flow rate of 50 µl.min^−1^. mIFNα2-V_H_D2 isotype control was also injected over the chip surface as a negative control for antigen binding.

When expressed as a genetic fusion to the C-terminus of mIFNα2, the DOM26h-196-61 dAb showed no reduction in affinity for ASGPR H1 as determined by surface plasmon resonance assays. K*_D_* of the unmodified dAb was determined to be 1 nM for the human antigen (data not shown) and 4 nM for the mouse antigen (Figure 3). K*_D_* of the mIFNα2-DOM26h-196-61 fusion protein for mouse ASGPR H1 was 0.6 nM indicating no detrimental effect on affinity of the dAb following fusion to mIFNα2.

### B16-Blue™ IFNα/β reporter cell assay

**Figure 4:**
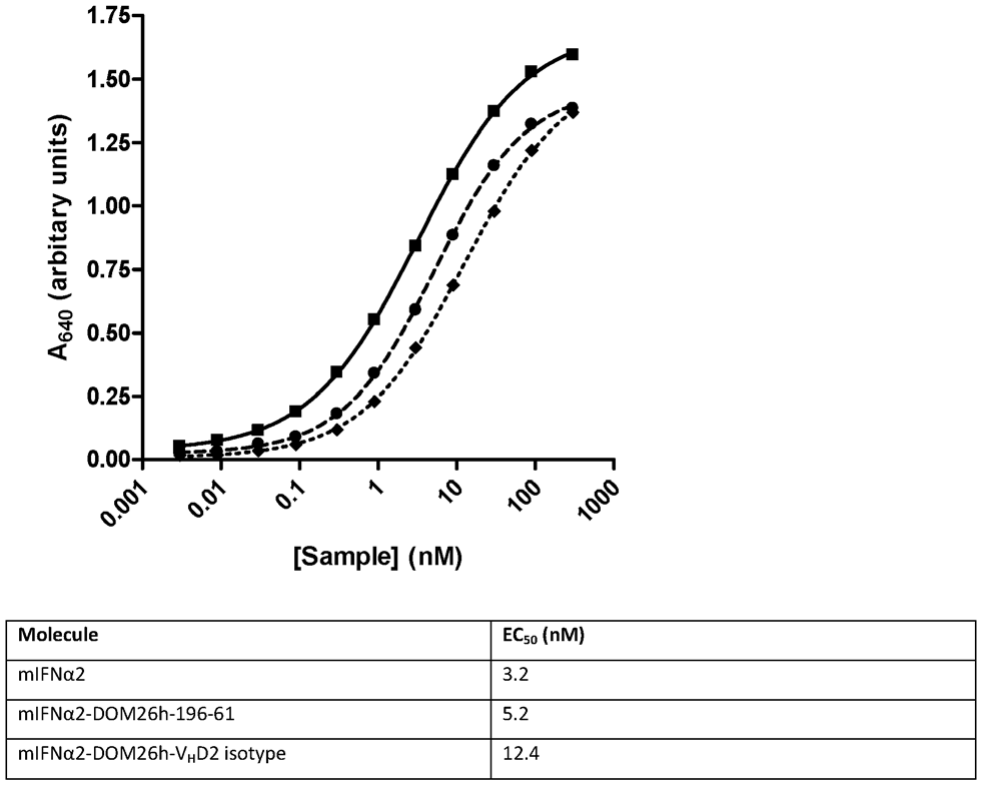
*In vitro* activity of mIFNα2 formatted as dAb fusions. Activity of the mIFNα2-dAb fusion proteins was tested in the B16-Blue™ assay and compared to unfused mIFNα2 standard. Error bars are not visible as they are smaller than the data points, but represent standard error of the mean of 3 independent experiments. mIFNα2-DOM26h-196-61 (dashed line, closed circles) and mIFNα2-V_H_D2 isotype control (dotted line, closed diamonds) showed comparable activity to the H_6_-mIFNα2 standard (solid line, closed squares), with only minor increases in the EC_50_.

The potency of mIFNα2 and mIFNα2-dAb fusion proteins was measured in the B16-Blue™ IFNα/β reporter gene assay (Figure 4). The potency of the DOM26h-196-61 and V_H_D2 isotype control mIFNα2-dAb fusions (EC_50_ = 5.2 and 12.4 nM, respectively) is similar in this assay to that of non-dAb fused mIFNα2 (EC_50_ = 3.2 nM). These data therefore show that dAb fusion to the C-terminus of mIFNα2 results in only a modest decrease in potency of the IFN fusion partner, in the range 1.6–3.9 fold in our experiments.

### Biodistribution and imaging of ^111^In-DOTA-DOM26h-196-61 and ^111^In-DOTA-V_H_D2

**Figure 5:**
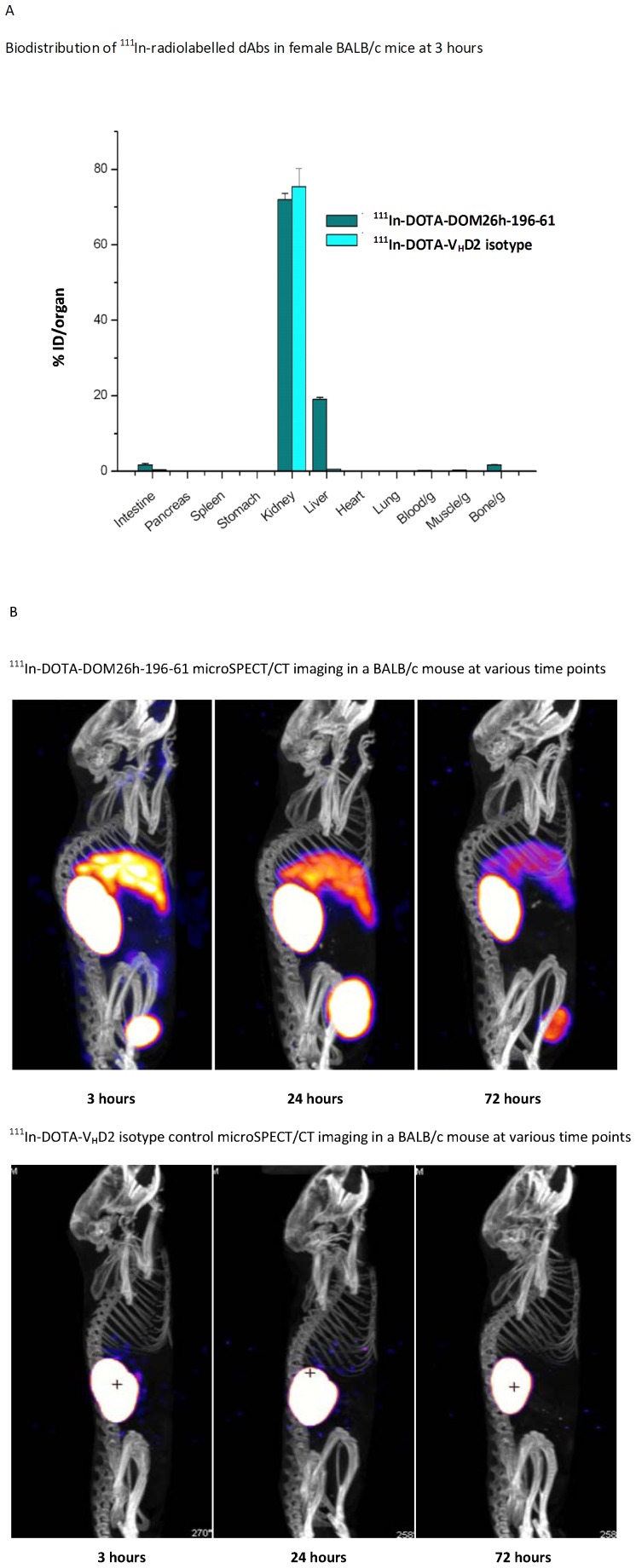
A – Quantitative analysis of ASGPR dAb biodistribution. Quantitative analysis of ^111^In labelled dAb levels was carried out 3 hours after intravenous administration in BALB/c mice via tail vein injection of approximately 0.5 MBq radiolabelled dAb. Results show accumulation of radiolabeled ASGPR dAb DOM26h-196-61 in mouse liver is considerably higher than that observed with isotype control dAb. By contrast minimal uptake of either ASGPR dAb DOM26h-196-61 or V_H_D2 isotype control dAb was observed in any other organ besides kidney. Error bars shown represent standard deviation of the mean, n = 4. **B**– *In vivo* imaging of ASGPR specific dAbs. Localisation of ^111^In labelled dAbs in BALB/c mice at 3, 24 and 72 hours post injection. Images were captured following intravenous administration of 14–15 MBq of radiolabeled dAb via tail vein injection in BALB/c mice. Images show that signal is observed in kidney and bladder with both dAb molecules, whereas liver localisation is only observed with anti ASGPR V_H_ dAb DOM26h-196-61.

DOTA conjugated dAbs were purified on protein A Streamline resin as described above, which provides a measure of protein functionality following conjugation. In addition DOTA conjugated DOM26h-196-61 was shown to be functional by BIAcore analysis of ASGPR binding activity (data not shown). Thin layer chromatography (TLC) analysis of the radiolabeled DOTA-DOM26h-196-61 and V_H_D2 isotype control dAbs showed efficient labelling of both antibodies, with minimal amounts (<1%) of insoluble particulates present after filtration (data not shown). Following tail vein injection of radiolabeled antibodies biodistribution studies showed that 19.1±0.6% of the injected dose (ID) of ^111^In-DOTA-DOM26h-196-61 remained in liver after three hours compared to only 0.55±0.07% ID/organ of the ^111^In-DOTA-V_H_D2 isotype control (Figure 5A). MicroSPECT/CT imaging studies of ^111^In-DOTA-DOM26h-196-61 confirmed uptake in liver with significant levels also observed in the kidneys, most likely due to renal clearance of the antibody (Figure 5B). No uptake was observed in any other tissue apart from bladder, consistent with renal clearance of radiolabeled DOM26h-196-61. In contrast, there was no visible uptake in liver of the V_H_D2 isotype control antibody. These data are therefore indicative of specific hepatic uptake of DOM26h-196-61 as a result of ASGPR binding *in vivo*.

### Biodistribution and Imaging of ^111^In-DOTA-mIFNα2, ^111^In-DOTA-mIFNα2-DOM26h-196-61 and ^111^In-DOTA-mIFNα2- V_H_D2 isotype control

DOTA conjugated mIFNα2 and mIFNα2-dAb fusions proteins were shown to be functional in the B16 assay, with DOTA conjugation apparently having minimal impact on EC_50_ values obtained in this assay. In addition DOTA conjugated mIFNα2-DOM26h-196-61 was shown to be functional by BIAcore analysis of ASGPR binding activity (data not shown). TLC analysis of radiolabeled DOTA-mIFNα2, DOTA-mIFNα2-DOM26h-196-61 and DOTA-mIFNα2-V_H_D2 isotype control showed efficient labelling of all three molecules, with minimal amounts (<1%) of insoluble particulates present after filtration (data not shown). When tested in the B16-Blue™ IFNα/β reporter gene assay the DOTA-conjugated mIFNα2, mIFNα2-DOM26h-196-61 and mIFNα2-V_H_D2 isotype control molecules were shown to retain interferon activity at levels comparable to the unconjugated proteins (data not shown). Similarly, SPR analysis of DOTA-mIFNα2-DOM26h-196-61 showed that the conjugate retained mouse and human ASGPR binding activity comparable to the unconjugated protein (data not shown) indicating minimal impact of DOTA conjugation on the affinity for antigen.

**Figure 6:**
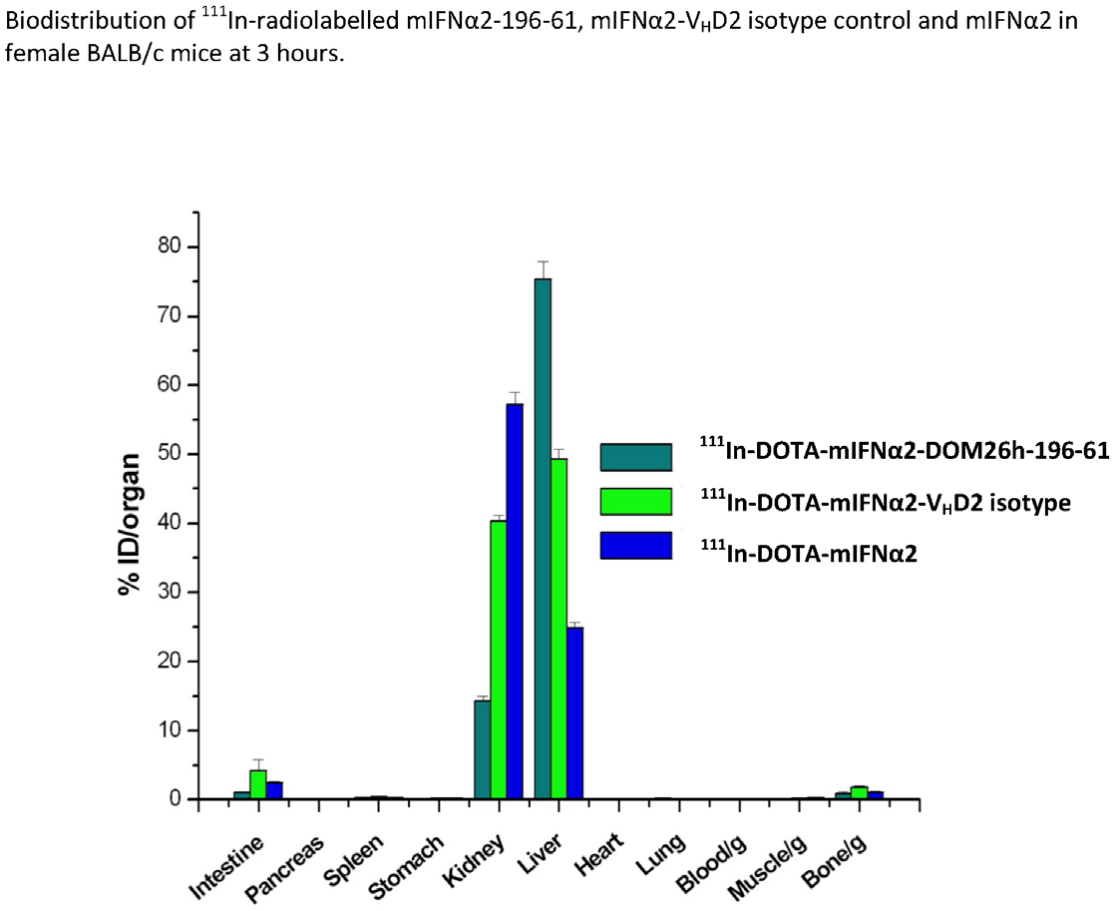
Quantitative analysis of mIFNα2 and mIFNα2-dAb biodistribution. Quantitative analyses of ^111^In labelled mIFNα2 and fusion protein levels were carried out 3 hours after intravenous administration in BALB/c mice via tail vein injection of approximately 0.5 MBq radiolabeled compound. Results show accumulation of radiolabelled mIFNα2-dAb fusions in mouse liver is considerably higher than that observed with mIFNα2. Data also shows increased hepatic accumulation of mIFNα2-DOM26h-196-61 compared to mIFNα2-DOM26h-V_H_D2 isotype control. Error bars shown represent standard deviation of the mean, n = 4 (n = 3 in the case of mIFNα2).

Following intravenous administration of radiolabeled compounds biodistribution studies showed that at 3 hours after injection significant hepatic uptake of both mIFNα2 and mIFNα2-V_H_D2 isotype control was observed, with 24.9±0.7% and 49.3±1.4% of the injected dose accumulating in liver respectively (figure 6). This is presumably due in part to binding of interferon receptor *in vivo* or hepatic clearance of mIFNα2, as the effect was observed in the absence of a dAb fusion partner. ^111^In labelled DOTA-mIFNα2-DOM26h-196-61 shows extremely high hepatic uptake, with 73.1±3.0% of the injected dose observed in liver at 3 hours after intravenous administration. Increased hepatic uptake of mIFNα2-dAb fusions compared to mIFNα2 alone may be due in part to the increased molecular mass of the fusion proteins, resulting in increased systemic exposure and delayed clearance, whereas the increased hepatic uptake of ^111^In-DOTA-mIFNα2-DOM26h-196-61 compared to that of ^111^In-DOTA-mIFNα2-V_H_D2 isotype control is most likely due to ASGPR binding of the fusion protein *in vivo*.

**Figure 7:**
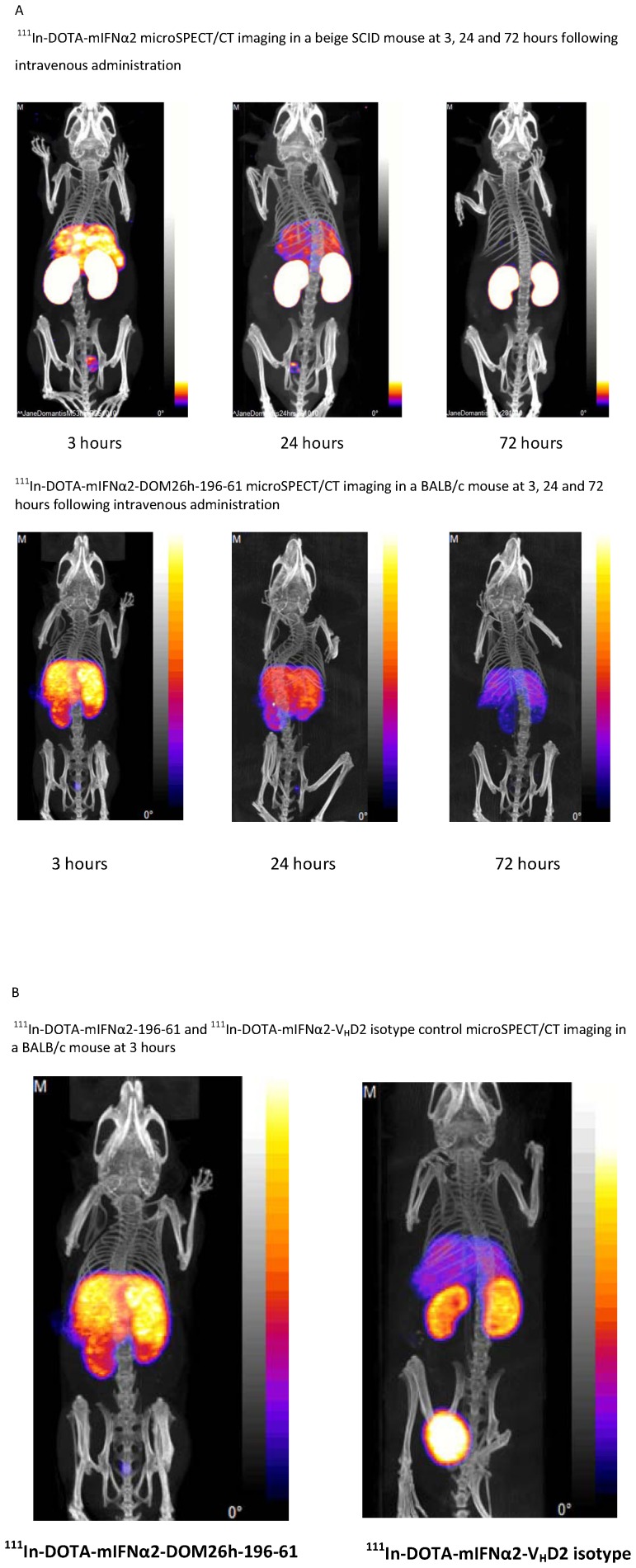
A – *In vivo* imaging of mIFNα2 and mIFNα2-DOM26h-196-61. Localisation of ^111^In labelled mIFNα2 in beige SCID mouse and ^111^In labelled mIFNα2-DOM26h-196-61 in BALB/c mouse at 3, 24 and 72 hours post injection. Images were obtained following intravenous administration of 12–15 MBq of radiolabeled compound via tail vein injection in BALB/c mice. Images show that signal is observed in liver, kidney and bladder with IFN molecules. **B**– *In vivo* imaging of mIFNα2-V_H_D2 and mIFNα2-DOM26h-196-61. Localisation of ^111^In labelled mIFNα2-dAb fusions in BALB/c mice at 3, 24 and 72 hours post injection. Images were obtained following intravenous administration of 12–15 MBq of radiolabelled compound via tail vein injection in BALB/c mice. Images show that signal is observed in liver, kidney and bladder with both fusion proteins; however the extent of liver uptake is clearly higher in the animal injected with mIFNα2-DOM26h-196-61, in agreement with biodistribution data.

MicroSPECT/CT imaging studies confirm uptake of ^111^In-DOTA-mIFNα2 in liver, with high levels in kidney and minimal uptake in all other tissues (figure 7A) which is interesting considering the ubiquity of IFNAR expression. By contrast the images obtained following ^111^In-DOTA-mIFNα2-DOM26h-196-61 administration shows higher signal in liver compared to kidney, reflecting the increased hepatic targeting properties of this molecule. It is clear, in agreement with the biodistribution data in figure 6, from comparison of the images of animals injected with ^111^In-DOTA-mIFNα2-DOM26h-196-61 and ^111^In-DOTA-mIFNα2-V_H_D2 isotype control that the liver-to-kidney ratio is higher in animals injected with the liver-targeted fusion protein (figure 7B), most likely as a direct result of ^111^In-DOTA-mIFNα2-DOM26h-196-61 binding to ASGPR expressed on the surface of hepatocytes.

## Discussion

Targeting of therapeutic payloads to specific cells or tissues is an attractive concept in terms of improving the safety and efficacy of the therapeutic. Antibody based methods for payload delivery have been described. For example, ASGPR single chain variable fragments (scFvs) conjugated to immunotoxins show increased cell killing in ASGPR expressing cell lines HepG2 and Huh7 compared to non-conjugated immunotoxin scFv fragments [27]. Here we demonstrate that domain antibodies specific to ASGPR expressed exclusively on hepatocytes can be used to target delivery of IFN to the liver.

Selection of ASGPR specific dAbs from phage libraries resulted in a number of distinct and sequence diverse dAbs with a wide range of affinities to the antigen. Following affinity maturation of ASGPR dAbs, clone DOM26h-196-61 was found to have high affinity for both human and mouse antigens, with 1 nM and 4 nM equilibrium dissociation constants observed when binding the human and mouse antigens respectively.

In order to assess binding to native ASGPR, which consists of H1 homodimers, H1/H2 heterodimers and tetrameric H1 complexes, flow cytometry assays were carried out using detection antibodies specific to human immunoglobulin heavy chain variable domains. We show that the ASGPR specific clone DOM26h-196-61 binds to primary human hepatocytes and the human hepatoma derived cell line Huh7, which has been shown to express ASGPR H1 subunit [29]. By contrast this antibody did not bind to non ASGPR expressing cell lines such as U937, and the V_H_D2 isotype control antibody did not bind to ASGPR positive cell lines including Huh7. Taken together these data provide evidence that DOM26h-196-61 binds specifically to cell surface expressed ASGPR H1 subunits, though it is not clear whether the antibody binds preferentially to distinct forms of ASGPR, for example the heterodimeric H1/H2 complex.

Given that our aim of developing ASGPR specific dAbs was to target therapeutically relevant payloads to the liver, we generated a genetic fusion of mIFNα2 and DOM26h-196-61 which could be easily expressed using the pTT-HEK mammalian cell culture system and purified by employing a simple affinity based capture method which utilises protein A coupled resins. The purified mIFNα2- DOM26h-196-61 fusion protein was subsequently shown to retain high levels of interferon activity in reporter gene assays and affinity for ASGPR in surface plasmon resonance analysis. ASGPR dAbs are therefore amenable to genetic fusion to interferon, without significantly affecting the activity of either fusion partner *in vitro*.

Natural ASGPR ligands have been shown to specifically accumulate in liver *in vivo* following, for example, intravenous administration of radio-iodinated asialorosomucoid in rats [30],^111^In labelled asialofetuin in mice and rats [31] and liposomes decorated with asialofetuin in mice [32]. Here we show, using microSPECT/CT imaging and biodistribution studies, striking differences between the distribution of the ASGPR specific dAb DOM26h-196-61 and the V_H_D2 isotype control dAb following radio labelling with ^111^In, with up to 20% of the injected dose of DOM26h-196-61 distributing to the liver 3 hours after intravenous administration. By contrast the V_H_D2 isotype control dAb shows minimal uptake in liver, with an apparent 40 fold reduction in liver uptake compared to animals injected with DOM26h-196-61. Levels of both antibodies were low in all other tissues assayed, with the exception of kidney and bladder. The significant amounts of radiolabelled DOM26h-196-61 and V_H_D2 isotype control dAb present in these tissues are not unexpected, as both antibodies are significantly below the molecular mass of approximately 66 KDa required to prevent renal clearance. Using immunohistochemistry with well characterised monoclonal antibodies to human ASGPR we saw no detectable expression in kidney, thyroid tissue or peripheral blood mononuclear cells, whilst demonstrating high levels of ASGPR expression in normal human liver tissue samples (data not shown). Hence, these microSPECT/CT studies conclusively demonstrate the liver targeting properties of ASGPR specific dAbs following systemic intravenous administration.

Imaging and biodistribution studies show that murine interferon alpha shows some degree of uptake in the liver in the absence of any fusion partner, and is perhaps not unexpected as this may reflect hepatic interferon receptor engagement responsible for driving the antiviral activity of interferon alpha *in vivo*. Alternatively these results may also be explained by hepatic clearance and metabolism. Fusion of murine interferon alpha to the V_H_D2 isotype control antibody resulted in elevated hepatic uptake compared to unfused interferon, which may in part be explained by the increased size leading to delayed clearance of the fusion protein, though detailed pharmacokinetic analysis will be required to confirm this. Fusion of murine interferon alpha to the liver-targeting dAb DOM26h-196-61 resulted in the highest level of hepatic uptake of any of the molecules studied in this example, approximately threefold greater than that observed with the unfused mIFNα2 protein. Despite the fact that the mIFNα2-V_H_D2 isotype control dAb and mIFNα2-DOM26h-196-61 fusion proteins were of comparable size, the hepatic uptake of mIFNα2-DOM26h-196-61 was significantly higher, leading us to the conclusion that this effect was a direct result of ASGPR binding on the surface of hepatocytes, and that ASGPR specific domain antibodies can be used to affect the biodistribution of therapeutically relevant payloads such as type I interferons, increasing the level of uptake in target organs and tissues. We therefore suggest that targeting IFN to the liver for the treatment of hepatropic virus infections such as HCV may result in increased efficacy through delivering the dose to the site of action but also that the dose of IFN could be reduced and the level of efficacy would be similar to the current standard of care. Either way we predict there would be improved safety implications following treatment with IFN.

There are documented examples of domain antibodies in development to address a number of autoimmune and inflammatory indications which would require repeat administrations of subcutaneously administered drug, for example TNF specific domain antibody constructs [33]. These molecules are currently undergoing evaluation in phase II clinical trials, demonstrating the utility of systemically administered domain antibodies in the development of novel therapeutics with antagonist activity. However as the approach described in this paper rather involves use of domain antibodies targeted to a specific tissue type in order to alter the distribution of agonist molecules *in vivo* this would merit further studies being conducted to investigate detailed pharmacokinetics and efficacy in preclinical models.
